# Novel Perspectives on the Relationship Between the Gastrointestinal Mucus Barrier and Soybean Agglutinin

**DOI:** 10.3390/cells15070620

**Published:** 2026-03-30

**Authors:** Tianjiao E, Jiajia Xia, Chengyu Xu, Xiapu Fan, Boyue Zhang, Nan Bao, Yuan Zhao, Guixin Qin, Yun Ji, Shumin Zhang, Saad Ahmed, Emad Mohammed Elken, Mohammed Hamed Eldawy, Li Pan, Mohammed Hamdy Farouk, Zhenlong Wu

**Affiliations:** 1 College of Animal Science and Technology, Jilin Agricultural University, Changchun 130118, China; etianjiao@mails.jlau.edu.cn (T.E.);; 2 State Key Laboratory of Animal Nutrition, Department of Animal Nutrition and Feed Science, China Agricultural University, Beijing 100193, China; 3 Animal Production Department, Faculty of Agriculture, Al-Azhar University, Nasr City, Cairo 11884, Egypt

**Keywords:** mucus barrier, mucin, soybean agglutinin, diseases, cancer

## Abstract

The gastrointestinal mucus barrier (GIMB) is a gelatinous structure consisting primarily of mucins, water, and cathelicidin. Such a structure is the first line of defense against pathogens in the intestinal cavity and acts an important environment for the survival and reproduction of symbiotic flora. Mucin is mainly synthesized and secreted by intestinal goblet cells, forming a slime layer with different structures throughout the intestinal tract. The process of mucin synthesis and secretion is regulated by many factors, and there are some differences in the physical and chemical properties of the GIMB among animal species. Furthermore, recent studies have shown a close relationship among the mucus barrier, gastrointestinal diseases, and tumors. Soybean agglutinin (SBA) is a major glycoprotein in soybean that is closely related with the detection, prevention, and treatment of disease and cancer. Current studies indicate a close relationship between SBA and the GIMB, particularly at the molecular level and through species-specific differences in mucin glycan structures. Existing evidence shows that these differences affect the binding affinity and antinutritional effects of SBA. The novel relations between SBA and GIMB may become new targets for disease treatment.

## 1. Introduction

The gastrointestinal mucosal barrier consists of the gastrointestinal mucus barrier (GIMB), the epithelial cell barrier (including intestinal epithelial cells as well as the cell top junction complex), and microorganisms [1]. The intestinal mucus layer serves as a primary defensive barrier that safeguards the gastrointestinal tract. Previously, the GIMB was considered a simple lubricant, promoting the excretion of food masses and intestinal feces, but now additional functions of the GIMB are gradually being revealed. The mucus barrier extends throughout the gastrointestinal tract, forming a protective layer that shields intestinal epithelial cells from exposure to exogenous harmful substances, digestive enzymes, and pathogenic bacteria [2,3]. This protection is critical to gastrointestinal health and function.

The GIMB mainly consists of mucins, water, inorganic salts, immune molecules, and antibiotic peptide. Mucins, which are highly glycosylated macromolecular proteins mainly secreted by goblet cells, are important components of the barrier. The synthesis of mucins usually occurs simultaneously with secretion and can be regulated by many factors [4,5].

Soybean agglutinin (SBA) is the most bioactive protein in soybean, accounting for 5~7% of soybean protein [6]. It is a glycoprotein that can specifically bind to N-acetyl-D-galactosamine, an amino sugar derivative of galactose, necessary for intercellular communication in both humans and animals [7].

Human and animal health can be significantly affected by the physicochemical properties of SBA [8], mucin [9,10], or their interaction. Accordingly, in the present review, we primarily focus on elucidating the types, structural features, biological functions, synthesis and secretion mechanisms of the mucin; describing the structural characteristics, antinutritional effects, and mechanism of SBA; summarizing the different influences of SBA and the GIMB on different animal species and their applications in the field of medicine; and emphasizing the relationship between the GIMB and SBA.

In recent decades, research on the GIMB and SBA has focused on each individually, with limited studies exploring their interplay and its effect of mucus barrier function. Systematic literature searches were conducted across PubMed, Web of Science, and CNKI databases, including unpublished studies. The final search was performed on 23 March 2026. Focusing on SBA, the mucus barrier, and the interaction between the two, a total of 150 relevant papers were selected to ensure the scientific rigor and comprehensiveness of this review.

## 2. Gastrointestinal Mucus Barrier

Mucus is a viscous, water-based material primarily composed of water (90–95%), electrolytes, lipids (1–2%), and proteins [11]. The main protein component of mucus is mucin, which is a large, highly glycosylated protein. In this section, we illustrate various aspects of the mucus layer that mainly depend on their structural units.

### 2.1. Composition and Structure of Mucus

Variations in the composition and structure of the mucus layer are observed along different segments of the digestive tract [12]. The thickness of the mucus layer varies in different intestinal segments. For instance, its thickness in mice reaches around 500 μm in the duodenum, 250 μm in the jejunum, 200 μm in the ileum, and 110–150 μm in the colon [13]. In humans, it reaches around 50–450 μm in the antral mucosa and 110 μm in the duodenum [14], whereas endoscopic biopsies of the distal colon show a total mucus thickness of 450 ± 70 μm [15]. Furthermore, the number of mucus layers differs among anatomical sites in various intestinal segments. The stomach is covered by an adherent mucus gel enriched in MUC5AC, which polymerizes into a mesh-like network that limits luminal hydrochloric acid diffusion, supports regulated acid handling, and protects surface epithelial cells from acid-mediated injury [11,16].

The intestinal mucus layer possesses a single layer of loose and permeable structure, allowing symbiotic bacteria to enter and contact the intestinal epithelium [17]. Thus, such bacteria are recognized by lymphoid antigen-presenting cells [18]. The antibacterial substances in the mucus layer can also isolate pathogenic bacteria from the upper epidermis [19].

The mucus of the large intestine is a double-layer structure with a loose and highly permeable outer layer and dense and tightly attached inner layer. The inner mucus layer is impermeable, while the outer mucus layer is large and highly permeable. This structure can be used as a habitat for symbionts. Compared with the intestinal cavity, the outer mucus layer has a unique microbial niche, and the microbial community shows different growth rates and resource utilization capabilities [20].

### 2.2. Main Characteristics of Mucin Protein

#### 2.2.1. Types of Mucins

Mucin is a macromolecular glycoprotein which mediates the formation and structure of mucus. Mucins are encoded by the MUC gene family and are grouped into two distinct forms, secretory and transmembrane. The types, expression, and biological functions of these two types of mucins are shown in Table 1 [21].

Secretory mucin is a skeleton mucin that forms a reticular mucus layer structure. This kind of mucin forms a large polymer through its N-terminal and C-terminal to protect the intestine from damage. This secretory molecule mainly includes large gel mucin (MUC2, 5AC, 5B, 6, and 19) and small soluble mucin (MUC7, 8, and 9) [22].

Transmembrane mucins include MUC1, 3A, 3B, 4, 11, 12, 13, 15, 16, 17, 20, and 21. There are many transmembrane mucins at the upper end of the intestinal epithelial cell membrane, and they can attach to it via a transmembrane domain. Transmembrane mucins present a large mucin domain at the N-terminus and a brief cytoplasmic domain at the C-terminus. On the outside of the membrane, some transmembrane mucins (MUC1, MUC3, MUC13, and MUC17) contain Sea urchin enterokinase agrin domain (SEA) regions, and some others (MUC4) possess NIDO-AMOP-v WD regions. Therefore, transmembrane mucins can be bound with each other by non-covalent bonds, and the extracellular region extends into the intestinal cavity [23,24]. Furthermore, enzymatic cleavage can mediate the release of transmembrane mucins from the plasma membrane while alternative splicing gives rise to secreted isoforms [25]. The types, expression site, and major biological functions of mucins are depicted in Table 1. 

#### 2.2.2. Structural Features of Mucin

Secretory and transmembrane mucins are highly glycosylated, and their sugar chains mainly include N-acetylgalactosamine (GalNAc), N-acetylglucosamine (GlcNAc), fucose (Fuc), galactose (Gal), sialic acid, and mannose [25]. The initiation of mucin biosynthesis is mediated by the polypeptide GalNAc-transferase ppGALNACT family which catalyze the addition of the first GalNAc carbohydrate unit to serine and threonine residues within target proteins [26]. There are about 20 homologous enzymes (inducing the same reaction) in human ppGALNACT. The basic structure of mucin is composed of peptide chain and GalNAc, which is called the Tn antigen [27]. This antigen is further structured by downstream glycosyltransferases to generate a series of core O-linked glycan polysaccharides [28].

**Figure 1:**
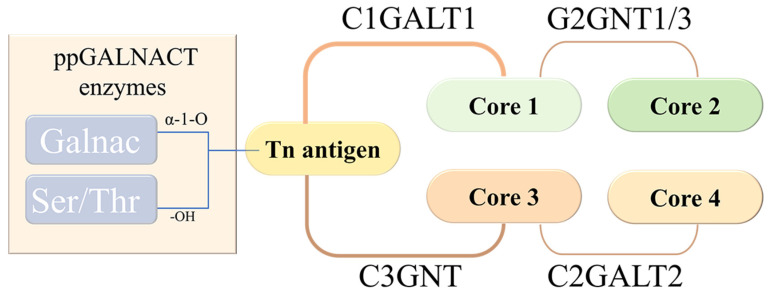
Structural features of mucin. O-glycosylation is initiated by the transfer of GalNAc to Ser/Thr residues, forming the Tn antigen, which is subsequently extended into Core 1, Core 2, Core 3, and Core 4 structures by distinct glycosyltransferases.

The core structure of the mucin O-sugar chain mainly includes Core 1, Core 2, Core 3, and Core 4 [29]. The starting point of its O-glycosylated sugar structure is GalNAc. Different glycosyltransferases are involved in the formation of these core structures. The glycosyltransferase enzymes include β1,3-galactosyltransferase (C1GALT1), β1,6-N-acetylglucosamine glycosyltransferase (C2GNT1, C2GNT2, C2GNT3, IGNT, C2GNT1, and C2GNT3), and β1,3-N acetylglucosamine transferase (C3GNT). These core structures can also add some sugar groups such as sialic acid, fucose, galactose, GalNAc, GlcNAc, and sulfate groups. Under the action of other types of glycosyltransferases, complex and diverse O-type sugar structures are formed [30], as shown in Figure 1.

Subsequent modifications of these core structures are conducted by other Golgi-localized glycosyltransferases to form complex O-linked glycans that participate in diverse biological processes [31,32].

There are many factors that regulate mucin synthesis and activity. For instance, the activation of peptide-based GalNAc transferase is not only controlled by the sequence background of the putative O-glycosylation sites [33] but also through epigenetic regulation, which can be controlled by competitive interactions among enzymes. The cell-specific O-glycosylation profile is ultimately shaped by the cellular repertoire of glycosyltransferases, their distinct specificities for sugar donors and acceptors, their ordered sequential reactions on structured platforms, and their differential localization within the Golgi subregion [34]. Furthermore, O-glycosylated mucin forms the skeleton structure of the mucus layer, which contributes to the viscoelasticity of mucus. Moreover, O-glycans protrude from the mucin protein core and closely interact with the extracellular surroundings [35]. Once the mucosal immune system has been damaged, the proportion and structure of the mucin core sugar chain in the small intestine and large intestine is damaged [36]. For example, the damage of the colonic mucus barrier in mice lacking the Core 1 structure leads to pathogens more easily passing through the protective barrier and approaching the intestinal epithelium, leading to spontaneous colitis [37]. The decrease in Core 2 sugar type level increases intestinal permeability and causes colitis (inflammation of the colon) [38]. When the expression of MUC2 and the number of goblet cells in mice lacking Core 3 sugar chain decrease, intestinal permeability is increased and bacterial translocation occurs [39].

O-glycans account for over 80% of the total molecular mass in mucins [40]. Such densely clustered O-glycan structures appear 10 to 100 times within individual mucin molecules and are commonly defined as tandem repeat regions [41]. Mucin-associated O-glycosylation exerts regulatory effects on multiple aspects of protein function. For instance, O-glycans can shield the polypeptide core from proteolytic cleavage and enhance protein stability against degradation [42]. They also adjust the serum half-life of chemokines and hormones to fine-tune their biological activity in vivo and mediate the intracellular transport of target proteins [43,44]. Moreover, mucin domains influence the aggregation behavior of membrane-associated glycoproteins, thereby regulating signaling activity and receptor binding capacity. Beyond protein functional modulation, mucin-type O-glycans act as specific recognition ligands for cell surface receptors and participate in the regulation of cell adhesion. Therefore, the activity of mucins is mainly affected by O-glycosylation and the integrity of the core structure.

#### 2.2.3. Mucin Biological Function in Disease Prevention, Control, and Treatment

Both secretory and transmembrane mucins maintain the integrity of the epithelial layer and other vital functions.

In the normal condition, mucin is secreted and located at the top edge of epithelial cells. However, during stress, the polarity of mucins is disrupted. Thus, apical proteins including transmembrane mucins undergo transient redistribution across the entire epithelial cell membrane [45]. This process enables interactions with cell surface molecules that are normally restricted to the basolateral membrane. Such redistribution of transmembrane mucins may lead to the loss of epithelial cell polarity. For instance, transmembrane mucins, especially MUC1 and MUC4, can influence the expression of tight junctions and adherens junctions [46,47]. Importantly, the abnormal expression and structural alterations of mucins can influence the progression of multiple cancer types [48,49].

Mucins also serve to restrict the activation of local inflammatory responses [50]. Accordingly, reduced mucin expression represents a critical connection linking inflammation to cancer development [51]. MUC2 deficiency promotes bacterial adhesion to epithelial surfaces, elevates intestinal permeability, and increases susceptibility to dextran sodium sulfate-induced colitis [52]. MUC2-knockout mice spontaneously develop colonic inflammation and superficial erosions similar to ulcerative colitis [53,54]. Among gel-forming mucins, MUC5AC and MUC6 are key mediators of mucosal defense and epithelial regeneration after injury in inflammatory bowel disease [55,56]. MUC13 also protects colonic epithelium by suppressing toxin-induced apoptosis, which may influence enteric infection, inflammatory bowel disease predisposition, and intestinal carcinogenesis [57,58]. Furthermore, the cysteine-rich domain of MUC3 participates in epithelial restitution and may serve as a novel candidate for intestinal wound healing therapy [59].

Mucin is also related to signaling pathways. Transmembrane mucins preserve the epithelial layer via signaling pathways that support cell proliferation and survival. MUC1 interacts with receptor tyrosine kinases in carcinoma cells, a process rarely observed in normal epithelial cells, and MUC4 enhances cell survival through diverse signaling routes [60,61]. An independent study reported that targeting MUC13 reduces liver cancer stem cell properties by blocking JNK/ERK pathway-dependent autophagy [62].

In these cases, mucins serve as both predictive markers for disease duration and promising therapeutic targets. Indeed, some mucins represent direct drug targets, and mucin inhibitors have been shown to suppress the survival and tumorigenicity of human tumors in experimental models [63,64,65].

### 2.3. Main Factors Affecting Mucin Synthesis

The intestinal mucus is mainly synthesized and secreted by epithelial goblet cells [66]. In the small intestine, the number of goblet cells in the crypt is greater than that in the villi. In the colon, the number of goblet cells at the top of the crypt is greater than that at the bottom.

There are particles filled with mucus at the top of the crypt, which are discharged by exocytosis after the particles mature [67]. The mucin monomer forms a dimer in the endoplasmic reticulum of goblet cells and is then O-glycosylated in the Golgi apparatus [11]. The release of intestinal goblet cell mucus particles is carefully designed. MUC2 and other mucus proteins accumulate in the secretory granules of vesicles [68] and are secreted in low-pH and high-Ca^2+^-concentration environments. The mucus secreted by intestinal goblet cells also contains other mucus components, such as TFF3 [69], CLCA1 [70], FCGBP [71], ZG16 [72], AGR2 [73], and RELM-β [74]. These proteins are crucial for preserving intestinal mucus barrier integrity and supporting gut health.

**Figure 2:**
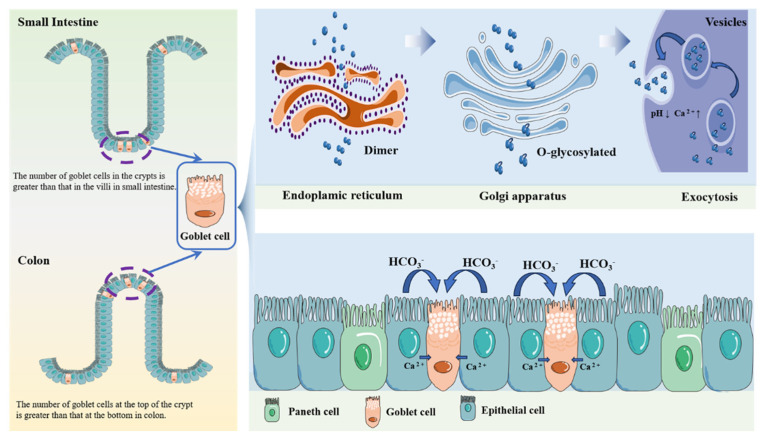
Distribution of intestinal goblet cells and main processes involved in mucin synthesis, maturation, and secretion. The figure shows regional differences in goblet cell localization in the small intestine and colon (**left**), as well as the intracellular pathway of mucin production and secretion, including dimer formation in the endoplasmic reticulum, O-glycosylation in the Golgi transport, and Ca^2+^ signaling to mucus granule expansion (**right**).

Mucin synthesis and secretion are generally coupled processes and are modulated by multiple regulatory factors. The mucus secretion mechanism of intestinal goblet cells depends on several cross-cellular processes, including endocytosis, autophagy, production of reactive oxygen species (ROS), ion concentration, and assembly and activation of inflammatory bodies, which jointly regulate the accumulation and secretion of mucus particles. Autophagy (for example, Atg5, Atg7, and LC3) defects in goblet cells reduce the production of mucin by affecting the production of ROS and the release of Ca^2+^ in ER [4]. Mucin secretion is controlled through an autophagy pathway involving amphisome formation via the fusion of autophagosomes and endosomes. This regulatory mechanism is dependent on ROS-mediated calcium homeostasis and can be accelerated during inflammation [75]. Wlodarska et al. [5] demonstrated that NLRP6 (nucleotide-binding oligomeric domain-like receptor protein 6) is a key coordinator of mucus secretion, and mice lacking NLRP6 exhibit mucus particle accumulation similar to Atg5/intestinal cells [4]. Ion secretion is essential for the formation of a functional small intestinal mucus layer [76]. In the small intestine, neighboring intestinal cells supply the goblet cells with the HCO3^−^ (a group required for mucin to unfold normally) [77]. Mice deficient in the functional cystic fibrosis transmembrane conductance regulator (CFTR), an ion channel responsible for Cl^−^ and HCO_3_^−^ secretion, exhibit elevated mucus adhesion and concentration in the digestive tract, which results in ileal obstruction [78]. The secretion of cell-like mucus granules is regulated by the Ca^2+^ concentration, and Ca^2+^ within intestinal cells signals to adjacent goblet cells through slit connections to complete the secretion and expansion of mucus [79] (Figure 2).

### 2.4. Characteristics of Digestive Tract Mucus in Different Animal Species

The characteristics of mucus are not identical among animal species. Looft et al. [80] analyzed the total protein mass in the mucus of different animals and found differences in the percentage of dry weight among different feeding animals, specifically, chickens (0.12%), turkeys (0.09%), pigs (0.08%), sheep (0.13%), and cattle (0.15%). The composition of mucus samples from different feeding animals is also different. For instance, sialic acids such as N-glycylneuraminic acid and N-acetylneuraminic acid are found in the glycans of cattle, pig, and sheep mucus, whereas chicken and turkey mucus contains N-acetylneuraminic acid as the sole sialic acid species. The thickness of the mucous layer in the digestive tract also is species-specific [81]. Mucus thickness in mice reaches 500 μm in the duodenum, 250 μm in the jejunum, and 200 μm in the ileum. In rats, however, the values are 170 μm, 124 μm, and 480 μm in the corresponding intestinal segments [82]. In pigs, the colonic mucus layer is approximately 100 μm thick, while the jejunal and ileal mucus layers are markedly thinner (<12 μm) [83]. In humans, the mucus layer is about 110 μm in the duodenum and approximately 450 μm in the colon [14,15].

The distribution of different core structure glycans in the digestive tract of animals may be affected by animal species, the intestinal segments of the digestive tract, and dietary composition [84]. The structure and composition of the mucus layer vary among animal species and across different body regions [85]. For example, human mucin exhibits a mesh size of 0.8 µm [86], whereas porcine mucin sheets have a typical pore diameter of approximately 200 nm [87]. Hence, there are differences in the total mass, composition, and physicochemical properties of proteins among the mucus of different animal species.

## 3. Research Progress on Soybean Agglutinin

SBA is a glycoprotein that can specifically bind with N-acetyl-D-galactosamine or galactose. The molecular weight of glycoprotein is about 120 kDa, the isoelectric point is 5.81, and the sedimentation coefficient is 6.0 s [88].

### 3.1. Structural Characteristics of SBA

**Figure 3:**
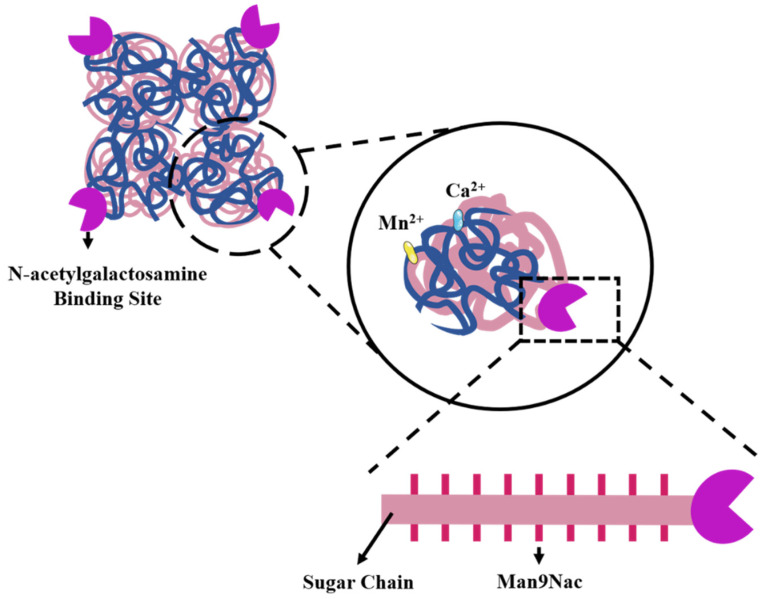
Structural characteristics of SBA. SBA is a tetrameric lectin. Each subunit carries an N-linked glycan and contains conserved Ca^2+^/Mn^2+^ binding sites. These sites are required for carbohydrate recognition.

SBA displays the typical four-domain architecture of legume lectins, consisting of four 30 kDa subunits [89]. The tetrameric three-dimensional structure of SBA exhibits a β-sandwich fold composed of two opposing curved 12-stranded β-sheets, forming a central cavity within the tetramer [90,91]. Each subunit of SBA possesses a covalently linked oligosaccharide chain, containing 9 mannose (Man9Nac). High-resolution nuclear magnetic resonance spectroscopy was used to characterize the basic structure of the sugar chains. The SBA molecule contains approximately 4.5% D-mannose and 1.5% N-acetylglucosamine, corresponding to 5 mol of glucose and 37 mol of mannose per mole of SBA. The glycan portion of SBA is covalently attached to the amide nitrogen of an aspartic acid residue in the polypeptide chain via an N-acetylglucosamine linkage [92]. Each SBA subunit also harbors tightly bound Ca^2+^ and Mn^2+^, which are essential for its carbohydrate-binding activity [93]. Furthermore, it binds to Mn^2+^ in a certain order, and then Ca^2+^ at other binding sites. The two binding sites must be in the optimal conformation state, as shown in Figure 3.

Biologists have detected the complete gene sequence of the soybean lectin subunit (gene library query numbers: K00821 and M30884) [94]. Such a sequence is deduced from the cDNA of soybean agglutinin as each subunit contains 253 base acid residues [95], which is lower than that determined by Lotan using the normal degradation method. In general, soybean lectin is deficient in cystine and low in methionine, but rich in acid and hydroxy amino acids, especially 4-hydroxyproline [95]. In glycosylated SBA, the oligosaccharide moiety contains 11 sugar residues [96]. The glycol-binding specificity of SBA is obtained by determining the degree to which glycologic components of known structures inhibit the biological activity of lectins. SBA has a separate sugar-binding site for each subunit, and each tetramer contains four N-acetyl-glucosamine binding sites [97]. De Boeck et al. [92] showed that the affinity of SBA for N-acetyl-glucosamine is 30 times compared to galactose. Such high affinity may be related to the fact that the specific binding of SBA to sugar is essentially the effect of hydrogen bonding. However, the 107 tyrosine of the soybean lectin subunit has an additional hydrophobic force with N-acetyl-glucosamine.

### 3.2. Antinutritional Effects of SBA on the Animal Digestive Tract

The main antinutritional effects of SBA are exerted on the digestive tract of animals. SBA is resistant to enzymatic degradation under both in vivo and in vitro conditions; residual SBA exists in the digestive tract of animals and has a certain impact on the structure and function of the digestive tract [98,99]. From a histological and pathological perspective, in the digestive tract, SBA causes different degrees of damage to the structure and function of gastrointestinal tissue. Fish fed with a diet containing SBA showed comparable pathological intestinal damage [100]. Elevated dietary soybean meal reduced muscularis thickness, fold height, and microvillus height in the distal intestine, while significantly upregulating the relative expression of IL-1β, IL-10, and IL-17F [101]. Excessive SBA intake causes oxidative stress and intestinal inflammation in rabbits [102]. When the concentration of SBA is too high, it causes systemic antinutritional reactions in animals, such as pathological changes in visceral organs and tissues, leading to intestinal flora disorder, diminishing the capability to trigger cell-mediated immune responses [103,104].

SBA can impair intestinal epithelial cell morphology, suppress cell viability and proliferation, and trigger apoptosis in monogastric IPEC-J2 cells [105]. These effects are mediated by cell cycle arrest at the G0/G1 phase, the downregulation of the tight junction proteins occludin and claudin-3, and the disruption of cell membrane permeability and integrity [106]. Soybean lectin may modulate the expression of genes encoding mediators that participate in inflammation-driven signaling pathways [107]. High-dose SBA can cause small intestinal microvillus atrophy in rats, reduce the viability of cells, and cause brush border membrane disorder [108]. Rats fed a diet containing 2 mg/mL SBA showed extensive shedding of epithelial cells into the intestinal lumen [109].

According to the results of whole-cell proteomic analysis, SBA-induced differentially expressed proteins are primarily enriched in pathways including DNA replication, base excision repair, nucleotide excision repair, mismatch repair, amide and peptide biosynthesis, and ubiquitin-mediated proteolysis, as well as mitochondrial and ribosomal structure and function [110]. Thus, the antinutritional mechanism of SBA is a complex process involving DNA-related events, protein synthesis and metabolism, signal transduction, and subcellular structural and functional homeostasis [110].

### 3.3. The Antinutritional Mechanism of SBA

Because the animal epithelium digestive tract is almost completely glycosylated [111], SBA binds specifically to intestinal epithelial cells. This binding effect is one of the pre-conditions and bases for SBA to produce toxic side effects and other antinutritional effects [105,112].

In the study of aquatic animals, SBA can bind to the intestinal epithelial cells of fish, but the main binding site is the posterior intestinal epithelial cells [113]. SBA can bind in many areas in the foregut and hindgut section of salmon, and as such these sections are more sensitive to SBA binding. This investigation indicated that after feeding with a diet containing a high level of soybean protein source (60%) and the same amount of purified SBA, there was a large number of SBA binding sites with the intestinal mucosa (especially the top of the fold) in rainbow trout and Atlantic salmon. Burrells et al. [114] found that dietary supplementation of 3.5% SBA leads to many SBA binding sites in the folds of the rear intestine of salmon.

Upon specific binding to the intestinal epithelial cell membrane, SBA alters cellular structure and function, thereby affecting the metabolic processes of intestinal epithelial cells. Histologically, when SBA specifically combines with digestive tract epithelial cells, the structure, function, and metabolism of animal digestive tract tissue and epithelial cells are changed, and the ability of intestinal digestion and absorption of nutrients is negatively affected. Gastrointestinal epithelial cytology results demonstrated that SBA specifically binds to the cytoskeletal protein α-actin in intestinal epithelial cells. This interaction indirectly modulates integrin gene expression and function, thereby influencing vital cellular processes including proliferation and apoptosis in IPEC-J2 cells [104,106]. In addition, SBA has been shown to affect the entry of Zaire ebolavirus into host cells [94].

### 3.4. Antinutritional Effects of SBA in Different Animal Species

The effects of SBA on animal growth performance depend on species, age, and administration dosage. For example, 50% of the growth inhibition effect of soybean on rats comes from SBA [115].

In rats, SBA bound to endothelial nectin-3 through terminal N-acetylgalactosamine residues [116], and it also suppressed weight gain in a dose-dependent manner [117]. Feeding broilers with diets containing SBA can cause intestinal epithelial hyperplasia and lead to abnormal development [118]. Moreover, the growth-depressing effects of SBA-containing diets in young animals are generally more pronounced in pigs than in chicks [119]. Sissons and Tolman [120] showed that SBA had no serious impact on the growth performance of ruminants.

The role of SBA among animal species also depends on specific intestinal segments. A higher residual rate of SBA was observed in the small intestine in sheep, followed by chickens, pigs, dogs, and rabbits [121]. There are also significant differences in the absorption and adsorption capacity of intestinal epithelial cells to SBA. The biological processes of SBA, such as adsorption, absorption, and degradation, mainly occur in the front of the small intestine in animals. Monogastric animals exhibit higher SBA absorption and adsorption capacity than ruminants. Among monogastric animals, rabbits have the strongest absorption capacity of SBA, chickens have weaker absorption capacity than rabbits, and the intestinal mucosa of pigs and dogs have more absorption capacity of SBA, and less absorption capacity. The sensitivity and tolerance of the intestinal mucosa to SBA differ among animal species. The sensitivity of the ruminant intestinal mucosa to SBA is low, while the permeability of the rabbit intestinal mucosa to SBA is the highest among monogastric animals. The trend in chickens and rabbits is essentially the same, and the absorption capacity of the rabbit is better than that of the chicken. The intestinal mucosae of pigs and dogs have a strong specific binding to SBA but weak permeability, which is more likely to produce antinutritional effects, caused by the adsorption of SBA on the intestinal tract [121]. There are also differences in the binding strength and degree of intestinal villi, crypts, and the whole intestinal segment with SBA in different animals. Hu [122] pointed out that the strongest binding strength of SBA to intestinal epithelial cells is located in dogs, followed by sheep, rabbits, chickens, and pigs. Moreover, SBA can cause differences in the changes in N-acetyl-D-galactosamine in the intestines of different animals. The content of N-acetyl-D-galactosamine in the intestines of pigs, rabbits, and chickens increases with the increase in dietary SBA concentration [123].

There are differences among animal species in the types of SBA-specific binding proteins on the erythrocyte membrane and intestinal epithelial cell membrane. Pan et al. [110] explored the mechanism underlying the species-specific differences in SBA-induced hemagglutination activity across rabbits, pigs, and calves. They found that SBA-specific binding proteins on erythrocyte membranes could be classified into three groups, namely, membrane skeletal proteins, catalytic enzymes, and functional regulatory proteins. The same protein is also detected in different animals, but most proteins exhibit species differences. SBA-specific binding proteins on intestinal epithelial cell membranes differ among animal species. The SBA-specific binding proteins of pigs are mainly distributed in PI5-8, with a molecular weight of 30–97.2 kDa. Similarly, those of chickens are mainly distributed in PI5-8, with a molecular weight of 29–64.2 kDa, and those of rabbits are mainly distributed in PI5-8, with a molecular weight of 26–90 kDa. The types and characteristics of the proteins are significantly different [123].

Therefore, among animal species, SBA exhibits effects on growth performance, its own kinetics, and the types of SBA-specific binding proteins on the erythrocyte membrane and the intestinal epithelial cell membrane.

### 3.5. The Role of SBA in Cancer Prevention, Diagnosis, and Treatment

SBA plays a positive role in cancer prevention. Through its interaction with cell surface glycans, it exhibits potential value in disease diagnosis. SBA can serve as a cancer biomarker to identify malignant tumor cells and facilitate cancer diagnosis and prognostic evaluation. For example, it has been applied to detect macrophages during the diagnosis of Whipple’s disease, which is a bacterial infectious disease affecting the digestive system [124]. SBA binding activity also acts as a valuable prognostic marker for colorectal cancer [125].

Interestingly, SBA can trigger cell death via autophagy and apoptosis, suggesting its potential involvement in cancer suppression. For example, it suppresses tumor cell proliferation in DL mice by inducing autophagic and apoptotic pathways. In vitro studies have revealed that SBA stimulates autophagic and apoptotic death in HeLa cells through ROS generation, and N-acetylcysteine (NAC)-mediated ROS inhibition reduces such cell death [126]. SBA may also serve as a differential agent for the selective removal of human breast cancer T-47D cells from tumor-contaminated bone marrow. SBA immobilized on magnetic beads provides a convenient approach for the ex vivo elimination of metastatic breast cancer cells in patients with advanced disease, and this strategy can be extended to other SBA-positive tumors [127]. Altered cell surface glycan expression in breast cancer is closely associated with tumor progression and metastasis, and such changes have been detected by specific SBA binding. SBA-conjugated silver nanoparticles also hold potential as nanocarriers for breast cancer therapy [128].

Although the application of plant lectins in cancer research remains at an early stage, advanced high-throughput techniques may promote the development of lectin-based complementary strategies for cancer management. Collectively, SBA displays notable preclinical and clinical value, highlighting its therapeutic promise in cancer intervention.

### 3.6. Current Methods for SBA Removal and Associated Limitations

Most lectins have relatively high thermal stability up to 70 °C and require strict heating conditions to be inactivated [107]. Due to the presence of numerous hydrogen bonds and hydrophobic bonds between the two monomers in the SBA molecule, SBA has a more stable structure and is less likely to lose its activity compared to other legume lectins.

Currently, thermal treatment is commonly used to eliminate the activity of SBA. SBA can be inactivated by wet heat treatment but not by dry heat conditions [89]. Generally speaking, the loss of sensory properties and nutritional value is caused by high-temperature heat treatment. Hyperthermal treatment effectively reduces SBA activity but can induce Maillard reactions between carbohydrates and basic amino acids like lysine. This process decreases free amino acid levels, lowers protein digestibility and nutritional value, and increases both energy consumption and processing costs [129]. SBA accumulation was effectively eliminated through CRISPR/Cas9-mediated editing of Le1 [130]. Soybeans can also be processed using non-thermal treatment methods such as ultra-high pressure. After processing soybeans at a pressure of 550 MPa for a certain period of time, the residual rate of active SBA is approximately 36%. However, the pressure has certain limitations on protein structure damage, and SBA may retain its original structural properties to a certain degree [131].

Therefore, given its incomplete inactivation under practical processing conditions, residual bioactive SBA is likely to reach the intestinal lumen after ingestion. As the mucus layer is the first physical and biochemical barrier encountered by luminal antigens, understanding how SBA interacts with the intestinal mucus and mucins is essential for explaining its effects on the intestinal barrier and for designing effective mitigation strategies.

## 4. Interaction Between SBA and GIMB

The interaction between SBA and the GIMB has become one of the research hotspots in intestinal biology and nutritional immunology in recent years. As the first line of defense against pathogens and harmful substances, the integrity of the intestinal mucus layer is essential for maintaining intestinal homeostasis. In this section, we will discuss the research progress on the interaction between SBA and GIMB, ranging from molecular mechanisms to species-specific differences.

### 4.1. The Molecular Mechanisms of SBA–GIMB Interaction

**Figure 4:**
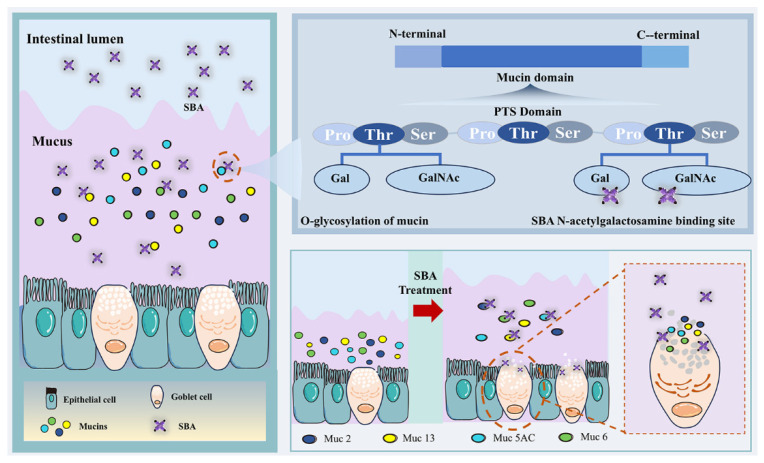
The relationship between soybean agglutinin and the gastrointestinal mucus barrier. SBA moves from the intestinal lumen to the mucus layer and binds mucin glycans, resulting in retention within the mucus (**left**). SBA preferentially recognizes terminal Gal/GalNAc on mucin O-glycans (**right**). This interaction alters mucus physicochemical properties and modulates goblet cell secretion (**right**).

The glycan-binding specificity of SBA provides the molecular basis for its interaction with mucins. SBA is a tetrameric lectin whose subunits each contain binding sites specific for galactose/N-acetylgalactosamine (Gal/GalNAc), showing a preferential recognition of GalNAc-containing oligosaccharide motifs [132]. This specificity enables SBA to bind selectively to mucins within the gastrointestinal mucus layer (Figure 4). The mucus layer is composed primarily of highly glycosylated mucins. MUC2, the major gel-forming mucin, is characterized by central mucin domains containing multiple tandem repeat sequences composed almost entirely of proline, serine, and threonine residues (PTS repeats). These regions serve as attachment sites for O-glycans, which are essential for conferring the gel-like properties of mucus [133]. For example, gastric mucus is produced by surface mucous cells (MUC5AC) and deeper glandular cells (MUC6), and O-glycan analysis shows predominant expression of the Core 2 motif [Galβ1–3(GlcNAcβ1–6) GalNAc-]. These glycans broadly contain galactose, GalNAc, and N-acetylglucosamine (GlcNAc), providing abundant potential ligands for SBA [35,113,134]. Based on these molecular recognition properties, the SBA–GIMB interaction follows a defined spatial pathway: SBA may bind specifically to the mucus layer before gaining access to the small intestinal epithelium to exert downstream effects. Such binding at the molecular level can directly alter the physicochemical properties of mucus, and it can also affect epithelial cell biology through signaling pathways and trigger cellular responses [135,136].

Beyond changes in the glycan pattern, the interaction between SBA and mucus also affects mucus thickness and goblet cell secretion. For example, SBA binding may induce the cross-linking or reorganization of mucin glycans, strengthening the mucus network’s mechanical properties and water retention and thereby increasing mucus layer thickness [137]. Using pig models, dietary inclusion of 0.2% SBA has been shown to modulate goblet cell function and mucus thickness through multiple mechanisms. SBA promotes goblet cell proliferation and enhances secretory activity, increasing goblet cell numbers in the villous region while leaving crypt numbers largely unchanged, which indicates region-specific effects on epithelial cell populations [138]. Importantly, SBA appears to alter mucus physical properties rather than simply increasing total mucin mass: SBA treatment has been associated with reduced measured levels of key mucins such as MUC2, MUC6, and MUC13 in intestinal tissues. This reduction may reflect accelerated secretion by goblet cells or the increased cross-linking density of mucins (via disulfide bond formation). Although MUC2 is the principal mucin in the intestinal mucus layer, other mucins (MUC5AC and MUC13) may compensate functionally by changes in glycosylation patterns or spatial distribution to preserve baseline mucus function. Moreover, combined proteomic and metabolomic analyses indicate that while SBA activates goblet cell function, it also stimulates the MAP2K6/p38 signaling pathway and induces intestinal oxidative stress (unpublished data).

### 4.2. Impact of Species-Specific Mucin Glycan Patterns on SBA Affinity and Antinutritional Effects

The mucus layers of different animal species show marked differences in glycosylation patterns, and these differences directly determine the affinity of SBA for the mucus barrier, thereby further influencing the intensity of its antinutritional effects in the intestine.

The structures of O-glycans in mucins differ among species. For example, bovine submaxillary mucin O-glycans are heavily modified with the sialic acid residues Neu5Ac and Neu5Gc and therefore exhibit a strongly acidic character, whereas the O-glycan profile of porcine gastric mucin is richer in neutral structural features, including N-acetylhexosamines, galactose, and fucose residues [139]. Such differences in glycosylation patterns directly affect the recognition and binding efficiency of SBA. As a lectin with specific affinity for N-acetylgalactosamine (GalNAc) and galactose residues, the interaction between SBA and mucins depends on the extent to which these glycans are exposed and on their structural presentation. Studies have shown that fucosylated glycans are present throughout the intestinal tract of pigs and rabbits, whereas fucose is absent from the chicken intestine. In contrast, the levels of N-acetylglucosamine and galactose glycosylation are higher in the chicken intestine than in pigs and rabbits. This indicates that the distribution of SBA-binding sites differs fundamentally among species. In chickens, the relatively high abundance of galactose residues may provide more direct binding targets for SBA, whereas in pigs, fucosylation may indirectly modulate SBA-binding efficiency by altering glycan conformation [80,123].

Differences in SBA’s affinity to bind to the mucus layer further contribute to differences in its antinutritional effects among species. Marked interspecies variation exists in mucin glycan structures and terminal modifications. Human mucins are predominantly enriched in sialylated glycans and also contain O-acetylated sialic acids and sulfated glycans. In contrast, rabbit and dog mucins are characterized by a high proportion of fucosylated structures, with only limited sialylation. Porcine gastric mucins can express sialidases, arylesterases, and sialic acid O-acetylesterases, which are capable of modifying and hydrolyzing glycan chains. Because the various modifications of sialic acids directly influence glycan recognition, they can substantially alter the binding efficiency and affinity of SBA [140,141,142].

SBA-binding epitopes are more highly exposed on mucins in poultry, allowing extensive binding and retention of SBA within the mucus layer. By contrast, in most mammals, sialic acid modifications can effectively mask these binding epitopes, enabling SBA to penetrate the mucus barrier more readily and reach the intestinal epithelial surface. However, SBA trapped in the mucus layer may still influence intestinal physiology. In vitro studies have shown that SBA can bind specifically to the rabbit jejunal mucosa and alter the uptake of glycoproteins by epithelial cells, suggesting that SBA may indirectly exert antinutritional effects by disrupting the diffusional and transport functions of the mucus layer [143]. These species-specific glycosylation patterns ultimately determine the adsorption, retention, and penetration of SBA within the intestine. In rabbits and chickens, the intestinal mucus exhibits relatively weak retention of SBA and higher mucosal permeability, thereby facilitating SBA absorption and leading to more pronounced antinutritional effects. In pigs and dogs, the intestinal mucus shows stronger adsorption of SBA but lower permeability, so its effects are exerted mainly through surface binding and localized action. Ruminants display weak recognition and binding of SBA due to their intestinal glycan modifications, conferring an overall higher tolerance to SBA [121,123].

Taken together, interspecies differences in mucin glycan composition and terminal modifications, such as sialylation, collectively determine the species-specific antinutritional effects of SBA in different animals by modulating processes such as binding and absorption.

### 4.3. Interactions Between Other Plant Lectins and the GIMB

Additionally, lectin–mucus interactions are supported by substantial evidence from other plant lectins. Plant lectins can recognize defined glycan structures, and mucins present abundant and diverse glycan modifications that enable direct binding under physiological conditions. Tomato lectin (TL), which binds GlcNAc, can recognize intestinal epithelial cells and shows affinity for Peyer’s patch-associated regions, and it has been exploited to enhance mucosal targeting in oral delivery systems [144,145]. TL has also been reported to bind the rat intestinal mucosa and to show partial resistance to intestinal digestive enzymes [146]. In addition, lectins such as Ricinus communis agglutinin (RCA), peanut agglutinin (PNA), Maackia amurensis lectin II (MALII), and Ulex europaeus agglutinin I (UEA) have been used as glycan probes to quantify interactions with purified porcine gastric and jejunal mucins, demonstrating glycan-dependent lectin–mucin binding that can be reduced by competitive sugars and that differs across mucins from distinct gastrointestinal regions [147]. Similar principles apply to hemagglutinin, where binding differences have been attributed mainly to specific terminal epitopes such as α1,2-fucosylation, illustrating how mucin glycosylation patterns can dominate recognition outcomes [148]. These findings collectively imply that mucin glycosylation heterogeneity is a central variable that may also shape SBA binding, but this remains to be tested directly.

### 4.4. Applications and Future Directions

We can use the close relationship between and similar characteristics of SBA and mucus to carry out relevant research work. In the middle of the 1900s, researchers determined the erythrocyte agglutination characteristics of mucin. The oligosaccharide antigens (A, GalNAca1-3(Fuca1-2) Galb1-4(3)GlcNAc; B, Gala1-3(Fuca1-2) Galb1-4(3)GlcNAc; and O(H), Fuca1-2Galb1-4(3)GlcNAc) that governed the ABO blood group specificities were also demonstrated to be attached to mucin scaffolds through GalNAc residues [149]. SBA’s basic biological function is to agglutinate animal red blood cells, and it possesses species-specific erythrocyte agglutination activity [150]. In addition, SBA and mucus play a certain role in disease prevention, detection, and treatment. Therefore, their combination may be effective in disease and cancer treatment. However, these hypotheses need to be verified by follow-up research.

## 5. Conclusions

The mucus layer of the digestive tract covers the surface of the epithelial cells, and the integrity is important as is the first line of defense in the gastrointestinal tract. Mucins are major macromolecular glycoproteins in mucus, synthesized and secreted by goblet cells; they are essential for maintaining the mucosal barrier. They are also highly glycosylated structurally, and their activity is mainly affected by O-glycosylation and the integrity of the core structure. SBA is a glycoprotein in soybean that can specifically bind to N-acetyl-D-galactosamine or galactose. This specific carbohydrate-binding ability underlies its close association with the gastrointestinal mucus barrier and is an important basis for its antinutritional effects. Molecular features and species-specific mucin glycan patterns are also important factors influencing its biological effects. Taken together, these findings have indicated specific mechanisms of antinutritional factors in food and feed, and provided new perspectives to investigate human digestive health and diseases, as well as related cancer treatments.

## Figures and Tables

**Table 1:** Features of different mucin types.

MUC Gene	Mucin Type	Expression	Biological Functions
MUC2	Secretory mucin	Small intestine and colon	Forms a physical barrier and protects epithelial cells from stress-induced damage. Provides attachment sites for the gut microbiota.
MUC5AC	Secretory mucin	Stomach—fundus	
MUC5B	Secretory mucin	Salivary glands, esophagus, stomach—fundus, small intestine, and colorectum	
MUC6	Secretory mucin	Stomach—fundus and small intestine	
MUC7	Secretory mucin	Salivary glands	
MUC8	Secretory mucin	Salivary glands	
MUC9	Secretory mucin	Small intestine	
MUC19	Secretory mucin	Salivary glands	
MUC1	Transmembrane mucin	Salivary glands, esophagus, stomach—fundus, small intestine, and colorectum	Contribute to the physical barrier. Transduce growth and survival signals into the cell.
MUC3A	Transmembrane mucin	Salivary glands, stomach—fundus, small intestine, and colorectum	
MUC3B	Transmembrane mucin	Salivary glands, stomach—fundus, small intestine, and colorectum	
MUC4	Transmembrane mucin	Salivary glands, stomach—fundus, small intestine, and colorectum	
MUC11	Transmembrane mucins	Small intestine and colorectum	
MUC12	Transmembrane mucin	Small intestine and colorectum	
MUC13	Transmembrane mucin	Esophagus, stomach, and colon	
MUC15	Transmembrane mucins	Small intestine and colorectum	
MUC16	Transmembrane mucin	Stomach—fundus and colorectum	
MUC17	Transmembrane mucin	Small intestine and colorectum	
MUC20	Transmembrane mucin	Colorectum	
MUC21	Transmembrane mucin	Colorectum	

## Data Availability

The original contributions presented in this study are included in the article. Further inquiries can be directed to the corresponding authors.
